# Transcriptomes of an Array of Chicken Ovary, Intestinal, and Immune Cells and Tissues

**DOI:** 10.3389/fgene.2021.664424

**Published:** 2021-06-30

**Authors:** Eliah G. Overbey, Theros T. Ng, Pietro Catini, Lisa M. Griggs, Paul Stewart, Suzana Tkalcic, R. David Hawkins, Yvonne Drechsler

**Affiliations:** ^1^Department of Genome Sciences, Interdepartmental Astrobiology Program, University of Washington, Seattle, WA, United States; ^2^College of Veterinary Medicine, Western University of Health Sciences, Pomona, CA, United States; ^3^Department of Genome Sciences, Department of Medicine, University of Washington, Seattle, WA, United States

**Keywords:** transcriptome, chicken, reproduction, intestinal cells, immunology

## Abstract

While the chicken (*Gallus gallus*) is the most consumed agricultural animal worldwide, the chicken transcriptome remains understudied. We have characterized the transcriptome of 10 cell and tissue types from the chicken using RNA-seq, spanning intestinal tissues (ileum, jejunum, proximal cecum), immune cells (B cells, bursa, macrophages, monocytes, spleen T cells, thymus), and reproductive tissue (ovary). We detected 17,872 genes and 24,812 transcripts across all cell and tissue types, representing 73% and 63% of the current gene annotation, respectively. Further quantification of RNA transcript biotypes revealed protein-coding and lncRNAs specific to an individual cell/tissue type. Each cell/tissue type also has an average of around 1.2 isoforms per gene, however, they all have at least one gene with at least 11 isoforms. Differential expression analysis revealed a large number of differentially expressed genes between tissues of the same category (immune and intestinal). Many of these differentially expressed genes in immune cells were involved in cellular processes relating to differentiation and cell metabolism as well as basic functions of immune cells such as cell adhesion and signal transduction. The differential expressed genes of the different segments of the chicken intestine (jejunum, ileum, proximal cecum) correlated to the metabolic processes in nutrient digestion and absorption. These data should provide a valuable resource in understanding the chicken genome.

## Introduction

In the United States, over nine billion broiler chickens, which is estimated to be about 19 billion kilograms of chicken products, are produced per year (NCC, 2019). Egg production totaled about 99.1 billion in 2019 in the United States (UEP, 2019). Apart from the important role in food production, the chicken has been used as an animal model to benefit key areas in functional human research including immunology (Glick et al., 1956), vaccine development (Matthews, 2006), reproduction (Nap et al., 2003, 2004; Bédécarrats et al., 2016), and nutrition (Klasing, 1984; Shang et al., 2018). The process to improve the annotation of the chicken is ongoing since it was first sequenced in 2004. As sequencing and data science technologies rapidly evolve, new tools allow for a more accurate representation of the chicken genome. The Functional Annotation of Animal Genomes (FAANG) project was launched to comprehensively characterize the genome of farm animals to address the sustainable agriculture of farmed animals (Giuffra et al., 2019). The current study under the FAANG project focuses on the accurate annotation of the coding and long non-coding (LNC) RNA transcripts of various cells and tissues.

The chicken karyotype consists of 38 autosomes and 2 sex chromosomes (Z and W). The first drafted chicken genome was sequenced using whole-genome shotgun sequencing of a female Red Jungle Fowl, which is the closest wild variant of the domestic chicken and was 1.05 Gb in length (Hillier LaDeana, 2004; Schmid et al., 2000). The current version of the chicken genome (Gallus_gallus-6.0; GCCA_000002315.5) was sequenced using the combined long single-molecule sequencing technology, and improved BAC and physical maps (Warren et al., 2017). This resulted in the increase of genome size to 1.21 Gb, accounting for micro-chromosomes that were not accounted for or incorrectly assembled in the previous version (Cheng and Burt, 2018). The coding and non-coding regions, as well as the regulatory elements, of the chicken genome is the current focus in annotation studies. Annotation of chicken genes is performed computationally from reference genomes of species that are better annotated. This method is successful in identifying conserved genes across species. However, it is challenging for non-conserved genes because of the relative physiology of the chicken compared to other species, in addition to different genome size, and differences in intron/exon organization between species (Shepard et al., 2009). Our annotation of the chicken genome has 16,779 protein-coding genes (28,345 transcripts) and 7,577 lncRNA and other RNA biotypes (10,943 transcripts). Of the 39,288 unique transcripts, 72.1% are protein-coding, 22.6% are lncRNAs, 2.9% are miRNAs and 2.4% are other RNA biotypes.

While the central dogma has established that coding RNAs are translated into proteins, there continues to be a growing interest in the function of ncRNAs, some of which are not transcribed by RNA polymerase II (Mattick and Makunin, 2006). Recently, it was discovered that ncRNA plays a regulatory role in many biological processes (Zhang et al., 2009). Long non-coding RNAs (lncRNAs), which are non-protein-coding RNAs more than 200 nucleotides in length, play a role in post-transcriptional epigenetic regulation (Quinn and Chang, 2016). In chickens, lncRNA regulates a host of biological functions, including intramuscular adipogenesis (Zhang et al., 2017a,b), sperm motility (Liu et al., 2017), cholesterol synthesis (Muret et al., 2017), and embryonic development (Roeszler et al., 2012). In Avian leukovirus-J (ALV-J) infection, lncRNA regulates macrophages by targeting genes involved in apoptosis, inflammation, and cytokine-cytokine interactions (Dai et al., 2019). A subtype of lncRNA, named long intergenic non-coding RNA, has been implicated in Marek’s disease (Han et al., 2017). Therefore, a comprehensive annotation of lncRNA expression in the chicken will reveal regulatory processes relevant to health and disease in an agriculturally important species.

In this study, we aimed to contribute to the catalog of transcriptomic differences of relevant chicken cells and tissues. We focused on multiple immune, intestinal, and reproduction-related tissues and cells. Specifically, tissue-specific immune cells (lung macrophage, spleen T cells, peripheral monocytes, and B-cells), immune organs (bursa and thymus), intestinal sections (jejunum, ileum, and proximal cecum), and ovary of the female reproductive tract were analyzed. The primary immune organs, the bursa, and thymus, are the origin of B cells and T cells in chickens, respectively (Cooper et al., 1966). The proximal cecum is located in the intestine at the ileocecal junction between the ileum and colon, is also the secondary immune organ in chickens due to the presence of mucosal-associated lymphoid tissues (MALT), such as the cecal tonsils. The findings described here will be useful toward a complete annotation of chicken tissue and cellular transcriptomes.

## Materials and Methods

### Experimental Animals

The animal procedure was approved and conducted according to guidelines established by the Western University of Health Sciences, Pomona, California (WesternU) Institutional Animal Care and Use Committee, protocol R17/IACUC/058. The F1 crosses of Line 6 and Line 7 from the Avian Disease and Oncology Laboratory (ADOL) were used in this study (Stone, 1975; Briles et al., 1977; Bacon et al., 2000). The two lines have identical major histocompatibility complex (MHC) B^∗^2 haplotype, but present different disease susceptibility to Marek’s Disease Virus (Line 6_3_: MDV-resistant and Line 7_2_: MDV-susceptible) (Liu et al., 2001). The F1 crosses of these lines have been used in other annotation studies by the FAANG consortium; therefore, it is used in this study to allow for a better comparison of the data. The chickens were held in open cages in the vivarium of the University Research Center at Western University. In addition to daily health monitoring, fresh food and water were provided *ad libitum*. Room temperature was adjusted to and maintained at 32°C until 3 weeks of age. To minimize the risk of pecking disorders, chicks were kept under restricted lighting conditions throughout the study. Peripheral blood was collected from jugular or wing web veins. Experimental animals were euthanized by insufflation of isoflurane.

### Sample Collection

All assays were performed in at least duplicates.

Immune tissue (thymus and bursa), intestinal tissues (jejunum, ileum, and proximal cecum), and reproductive tissue (ovary) were collected and flash-frozen in liquid nitrogen for later use. Tissue immune cells (lung macrophage, and spleen CD3+ T cells) were collected from the organs homogenized and filtered through 70 μm nylon cell strainers.

Tissue macrophages and T cells were extracted using magnetic beads (Dynabeads FlowComp Flexi, Invitrogen, Carlsbad, CA, United States) coated with biotinylated-mouse-anti-chicken-monocyte/macrophage-monoclonal antibodies (Clone KUL-1, Cat. No. 8420-08, SouthernBiotech) and biotinylated-mouse-anti-chicken-CD3-monoclonal antibodies (Clone AV-20, Cat. No. 8200-08, SouthernBiotech, Birmingham, AL, United States), respectively.

Peripheral blood B cells were collected from the blood (Collisson et al., 2017). Briefly, the blood was diluted in an equal volume of PBS and layered slowly over Ficoll-Histopaque (1.083 g/mL) (Sigma-Aldrich, St. Louis, MO, United States), and then centrifuged for 35 min (400 × *g* at 23°C with the brake off). The interface containing the peripheral blood mononuclear cells (PBMCs) and B cells were collected. Peripheral blood B cells were extracted using magnetic beads (Dynabeads Pan Mouse IgG, Invitrogen, Waltham, MA, United States) coated with unlabeled-mouse-anti-chicken-Bu-1a/b-monoclonal antibodies (Clone AV-20, Cat. No. MCA5764, Bio-Rad, Hercules, CA, United States). Peripheral blood monocytes were collected from PBMCs. Briefly, after density gradient separation using Ficoll-Histopaque as described above, the monocytes were extracted from the PBMC using magnetic beads coated with unlabeled-mouse-anti-chicken-KUL01 monoclonal antibodies (SouthernBiotech).

The metadata and associated protocols concerning the 20 tissues have been deposited in the Biosamples database with the identifiers SAMA8868413 to SAMA8868433.

### RNA Extraction and Library Construction

Total RNA from tissues and immune cells was collected using a modified Trizol/Chloroform method. Briefly, a second chloroform phase extraction and a second ethanol wash were included in the modified method. The total RNA from tissues was purified, and DNase treated using the Direct-zol RNA Miniprep Plus (Zymo Research, Irvine, CA, United States). The total RNA from immune cells (lung macrophage, spleen T cells, peripheral blood B cells, and peripheral blood monocytes) were not purified using the Direct-zol RNA miniprep Plus due to the lower concentration of the immune cell RNA compare to the tissue RNA. The total RNA from immune cells was DNase treated after extraction. Quality control of the total RNA was performed fluorometrically using the Qubit RNA HS Assay Kit and Qubit 3 (Thermo Fisher Scientific, Waltham, MA, United States) and RNA 6000 Nano Kit and Bioanalyzer 2100 (Agilent, Santa Clara, CA, United States). Total RNA with RNA integrity number (RIN) above 8.0 were used in the stranded library generation process using the Zymo-Seq RiboFree Total RNA Library Kit (Zymo Research). ERCC RNA Spike-In Controls (Invitrogen) were used to create a standard baseline measurement of RNA. Ribosomal-RNA (rRNA), globin, and overrepresented transcripts were removed, and sequencing adaptor ligation of the cDNA was removed by size selection and PCR enrichment. Libraries were barcoded with P5 and P7 index sequences according to the manufacturer’s protocol.

### RNA-Sequencing

Libraries were pooled and sequenced on HiseqX-PE150 by Novogene Bioinformatics Technology Co. (Beijing, China). Libraries were sequenced to an average depth of 43.7 million paired reads per library.

### Bioinformatics Analyses of RNA-Sequencing Data

Raw reads were trimmed with TrimGalore (v0.4.1, parameters: –clip_R2 2) (Martin, 2011). Trimmed reads were mapped and quantified using STAR (v2.6.1c) and RSEM (v1.3.1) using the function rsem-calculate-expression (parameters: –star –sort-bam-by-coordinate) and the reference file Ensembl annotation release GRCg6a, Ensemble annotation release 98, genome-build-accession NCBI:GCA_000002315.5 (Li and Dewey, 2011). Read counts (raw, trimmed, aligned) can be found in Supplementary Table 1. Transcriptomes were assembled using StringTie (v2.1.4) and gffCompare (v0.11.6, parameters -R -r) (Pertea and Pertea, 2020). Counts of genes and transcripts from Figures 2, 3B,C were obtained from the output of gffCompare.

Euclidean distance, pairwise correlations, and PCA plots were generated by pcaExplorer (Marini and Binder, 2019). PCA was performed using all expressed genes, used the gene counts from the RSEM quantification, and the gene counts were first normalized with DESeq2 (v1.30.0) (Love et al., 2014). Heatmaps were generated with Morpheus^1^ (Gould, 2016). Shannon’s entropy calculations were performed with the BioQC function entropyDiversity (Zhang et al., 2017a). Count matrices inputted to BioQC were normalized with DESeq2 and used counts from the RSEM output. Isoform entropy had an additional filter, requiring that the isoform’s gene be expressed in at least two cell types. For all analyses, isoforms were considered expressed if they had an average TPM greater than 0.5 across replicates from the RSEM quantified counts were included. Sashimi plots were generated with ggsashimi (parameters: -M 10 -C 3 -O 3 –shrink –alpha 0.25 –base-size = 20 –ann-height = 4 –height = 3 –width = 18) (Garrido-Martín et al., 2018). Browser shots were generated using the UCSC genome browser (Kent et al., 2002). BigWig files for the UCSC genome browser were generated from the mapped bam files using deepTools bamCoverage (v3.5.0) (Ramírez et al., 2014). Transcription start site (TSS) annotations for head-to-head (H2H) detection was obtained from the UCSC table browser using the settings “clade: Vertebrate,” “genome: Chicken,” “assembly: Mar. 2018 GRCg6a/galGal6,” “group: Genes and Gene Predictions,” “track: Ensembl Genes,” and “table: ensGene.”

Extended lncRNA analysis was performed using the annotation from Jehl et al. (2020)^2^ (LNCextendedEns101.gtf.gz). Reads were pseuo-aligned to this reference first be converting the reference to a fasta file with gffread. Then a kallisto index was generated with kallisto index (parameter: –make-unique) and sample TPMs were obtained with kallisto quant. A TPM > 0.5 was used for an expression threshold. BioQC entropyDiversity was used to calculate the most specific lncRNAs by tissue type.

Differential gene expression was calculated using DESeq2 (v1.30.0) (Love et al., 2014). Genes with an adjusted *p*-value less than 0.05 were considered differentially expressed. GO biological processes were calculated using WebGestalt (Liao et al., 2019) with an FDR threshold of 0.05 for determining GO category overrepresentation. WebGestalt was run with the basic parameters “Gallus gallus,” “Over-Representation Analysis (ORA),” “Gene Ontology, and “Biological Process.” “genome” was selected as the reference set. Figures 5C, 6C and Supplementary Figure 3B directly use these GO terms. Figures 5B, 6B display the weighted set cover, which reduces redundancy of the categories displayed. Full GO categories corresponding to the weighted set covers are provided in Supplementary Tables 5, 6. Venn diagrams were generated with Intervene (Khan and Mathelier, 2017). All tools used the default parameters unless otherwise indicated.

## Results

### Sample Clustering and PCA

**FIGURE 1:**
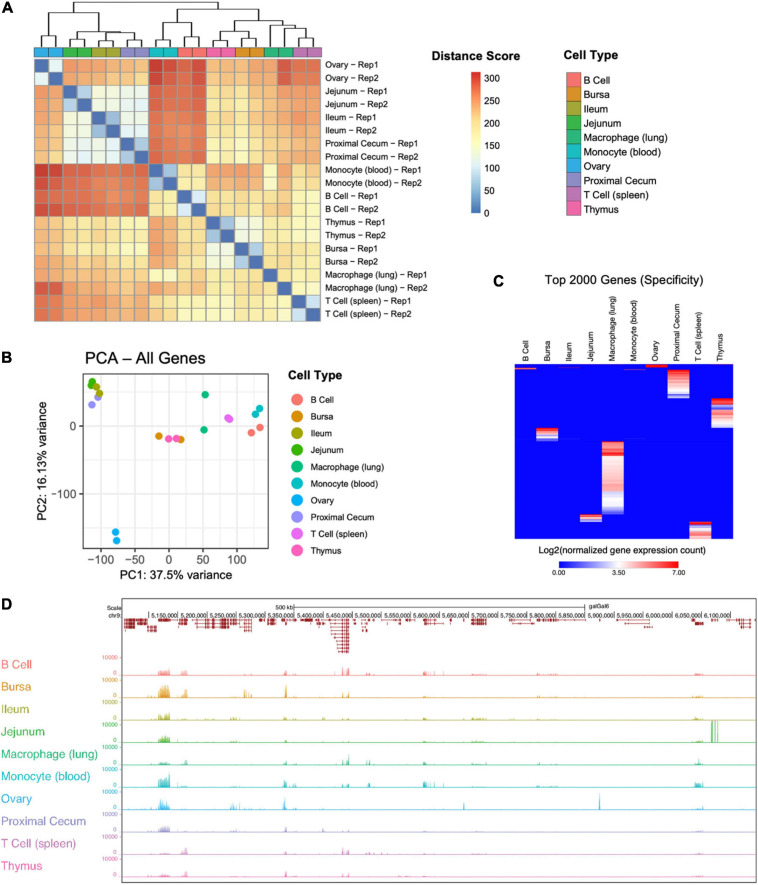
Overview of tissue RNA-sequencing results. **(A)** Sample to sample distance heatmap quantifying the Euclidean distance between each sample. **(B)** Principal component analysis using all genes for all samples. **(C)** Expression of the 2000 genes with the highest Shannon’s entropy values. Rows sorted using Euclidean distance. **(D)** UCSC browser shot of RNA-seq data showing variable expression among samples.

Ten cell and tissue types were profiled with RNA-sequencing with the goal of determining coding and primarily lncRNA expression, as well as isoform usage. All samples were compared to one another using Euclidean distance (Figure 1A) and principal component analysis (PCA) (Figure 1B) using the R package pcaExplorer (Marini, 2016). Replicates of the same tissue had the smallest Euclidean distance between one another (Figure 1A) and the highest Pearson correlation scores, except for the macrophages that seem to be somewhat distant in the second PCA dimension, and the highest Pearson correlation scores (Supplementary Figure 1). All expressed genes (Figure 1B) were used for PCA. Samples appear to form three distinct clusters based on functional category: immune system [B cells, bursa, macrophage (lung), monocytes (blood), T cells (spleen), thymus], reproductive tissue (ovary), and intestinal tissue (jejunum, ileum, proximal cecum). To identify genes highly specific to tissue or cell types, Shannon’s entropy was calculated for each gene across all cell types, obtaining a specificity score for each gene. The expression of the 2000 most specific genes was visualized in a heatmap (Figure 1C), revealing that macrophage cells have the most specific gene expression, while ileum tissue and monocytes have the least. When the next 2000 most specific genes are visualized (Supplementary Figure 2A) we begin to see less tissue-specific expression and see genes that are expressed in a small subset of cell types, compared to the 1000 least specific genes (Supplementary Figure 2B), which show more uniform gene expression across all tissue and cell types. A UCSC browser shot of gene expression across all cell and tissue types shows the uniformity of expression among some genes and variable expression among others (Figure 1D).

### Transcriptome Coverage and Biotype Detection

**FIGURE 2:**
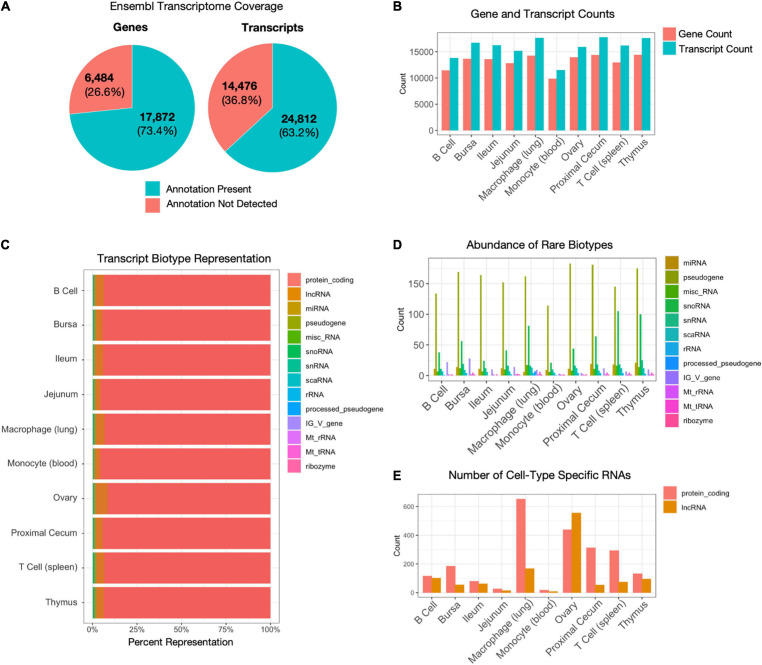
Gene and transcript characterization. **(A)** Percentage of annotated chicken genes and transcripts detected across all samples. There are 24,356 genes and 39,288 transcripts in the *Gallus gallus* GRCg6a Ensembl annotation. **(B)** The number of genes and transcripts detected per cell type. Gene counts range between 9,839 [monocyte (blood)] – 14,418 [thymus]. Transcript counts range between 11,522 [monocyte (blood)] – 17,794 [proximal cecum]. **(C)** Breakdown of transcript types detected per cell type, by percentage. **(D)** Counts of low abundance transcript biotypes with less than 3% representation (all transcript biotypes, except lncRNA and protein-coding RNA). **(E)** The number of protein-coding RNAs and lncRNAs unique to each sample type.

Among all samples, 73.4% (17,872) of all known chicken genes and 63.2% (24,812) of all known transcripts from Ensembl annotations were detected (genome build GRCg6a) (Figure 2A). Tissue and cell type-specific gene, transcript, and lncRNA counts are provided in Table 1. Between 9,839 (monocyte) – 14,418 (thymus) genes and 11,522 (monocyte) – 17,794 (proximal cecum) transcripts were detected in each sample (Figure 2B). Out of the fifteen transcript biotypes (protein_coding, lncRNA, miRNA, pseudogene, misc_RNA, snoRNA, snRNA, scaRNA, rRNA, processed_pseudogene, IG_V_gene, Mt_rRNA, Mt_tRNA, ribozyme, sRNA) in the *Gallus gallus* reference annotation, fourteen were found in each of the sample types. The largest number of transcripts detected was from protein-coding RNA and lncRNA (Figure 2C). Among all samples, 28,345 (90.0%) protein-coding transcripts were detected. More recently, an extended lncRNA annotation was released (Jehl et al., 2020). For this extended analysis, we used the genome annotation file for LNC-enriched Ensembl RNAs, which showed that 3,723 lncRNAs were identified among all cell and tissue types. Even though our library preparation method did not enrich for small RNAs, a low level of these transcripts was detected (Figure 2D). Additionally, protein-coding and lncRNA expression unique to each cell or tissue type was detected (Figure 2E and Supplementary Table 2). All cell and tissue types had a greater number of unique protein-coding genes, except for the ovary tissue, which had a higher number of unique lncRNAs. Lung macrophage expressed the most unique protein-coding genes (653), whereas jejunum tissue (28) and monocytes (19) expressed the fewest. For jejunum tissue, this may be attributable to the fact that other intestinal tissues, the proximal cecum and the ileum, were included in the analysis and may have more similar gene expression profiles than other tissues included in this study. The number of lncRNAs per tissue ranged from 464 [monocyte (blood)] to 2,179 [macrophage (lung)] (Supplementary Figure 3A and Table 1). Many of these lncRNAs were specific to a single tissue, with tissue-specific lncRNAs ranging from 4 [monocyte (blood)] to 408 [macrophage (lung)] (Supplementary Figures 3B,C and Supplementary Table 2). Since we did not sequence samples to a depth of 100 million aligned reads as recommended by FAANG for novel gene annotation, we did not attempt to discover new genes.

**TABLE 1:** The number of transcripts, genes, and lncRNA by tissue.

Type	Tissue	# of	# of	# of	Extended lncRNA
		transcripts	genes	lncRNA	analysis #
**Immune**	B cell	16,170	12,945	648	958
	Bursa	13,825	11,442	608	1,433
	Monocyte (blood)	17,794	14,380	316	464
	Macrophage (lung)	11,522	9,839	868	2,179
	T cell (spleen)	16,703	13,642	731	1,630
	Thymus	17,639	14,240	757	1,718
**Intestinal**	Jejunum	15,172	12.820	398	1,311
	Ileum	15,924	13,940	710	1,007
	Proximal cecum	17,619	14,418	690	1,746
**Reproductive**	Ovary	16,255	13,585	1,063	925

### Isoform Characterization

**FIGURE 3:**
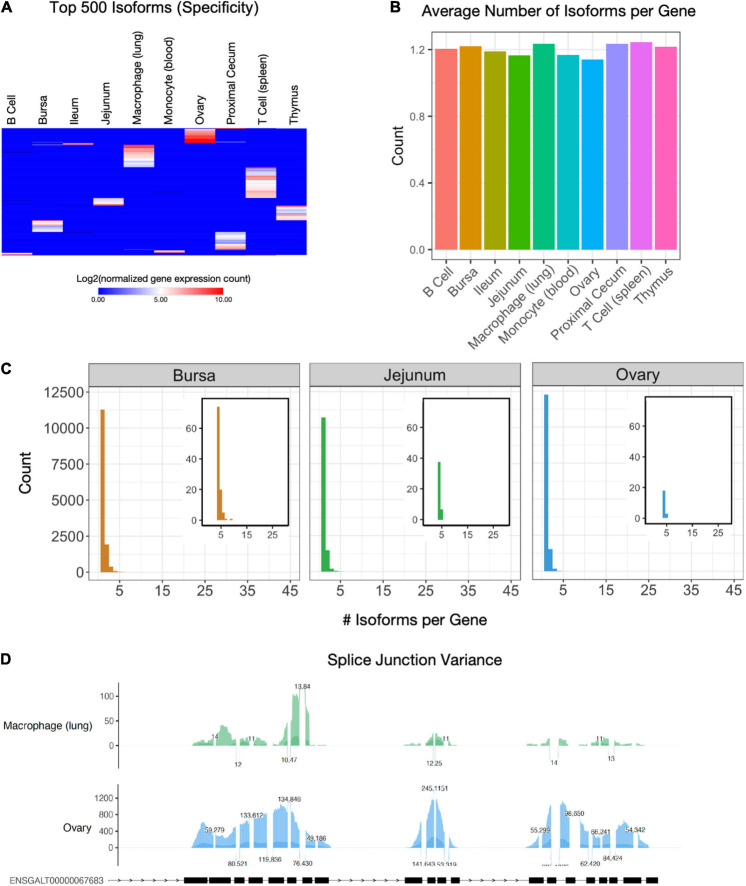
Isoform characterization. **(A)** Expression of the 500 isoforms with the highest Shannon’s entropy values. Rows sorted using Euclidean distance. Isoforms have been filtered for genes that have a TPM of at least 0.5 in at least two cell types. **(B)** The average number of isoforms per gene for each cell type. **(C)** Histogram of isoform counts per gene. The cutout plot in the upper-right corner is a zoomed-in section for 4+ isoforms per gene. **(D)** Sashimi plots of splice junction variance for macrophage (lung) cells and ovary tissue for gene *PDGFRB* (ENSGAL00000030613), which has a single annotated transcript ENSGALT00000067683.

Alternative splicing is a primary mechanism for diversifying protein expression. After constructing transcript isoforms from short-read sequencing, Shannon’s entropy calculations revealed unique isoforms to each cell and tissue type were found among a set of 500 isoforms (Figure 3A and Supplementary Table 3). The highest number of unique isoforms was found in T cells. The lowest was in monocytes, B cells, and ileum tissue. When expanded to view expressions of the top 1,000 isoforms with the highest specificity, isoforms are less specific to a single cell or tissue type (Supplementary Figure 2C). In contrast, when the 1,000 isoforms with the least entropy are observed, we see uniform expression among most cell and tissue types (Supplementary Figure 2D). Each cell and sample type has an average of 1.14 (ovary) – 1.24 (spleen T cell) isoforms per gene (Figure 3B). Histograms allow us to further visualize the distribution of isoform counts per gene in each tissue (Figure 3C and Supplementary Figure 4A). Most genes express only a single isoform of around 10,000 for each cell and tissue type. Between 1396 (blood monocyte) – 2667 (proximal cecum) genes per cell type express two isoforms. A small subset of genes expressed more than four isoforms of a gene (Figure 3C, Supplementary Figure 4A insets, and Supplementary Table 3). There were 204 genes with four or more isoforms expressed among all cell and tissue types. The gene with the most isoforms is ST6GAL1, which has 10 isoforms in spleen T cell tissue. They fall into the GO biological process categories “localization within membrane,” “activated T cell proliferation,” and “cell migration” and the GO molecular function category “kinase binding” (Supplementary Figure 4B). To visualize differential splice junctions, a sashimi plot was generated for each sample (Figure 3D and Supplementary Figure 5A) for the gene *PDGFRB* (ENSGAL00000030613). Bursa, ileum, jejunum, ovary, proximal cecum, and thymus tissue express nearly all exons, whereas B cells, macrophages, monocytes, and T cells express a subset of exons. A UCSC browser shot of the gene *PDGFRB* (ENSGAL00000030613) also assists visualization of these differences in isoform expression of a single gene among different tissue and cell types (Supplementary Figure 5B).

### Co-expression and Mono-Expression on Forward and Reverse Strands

**FIGURE 4:**
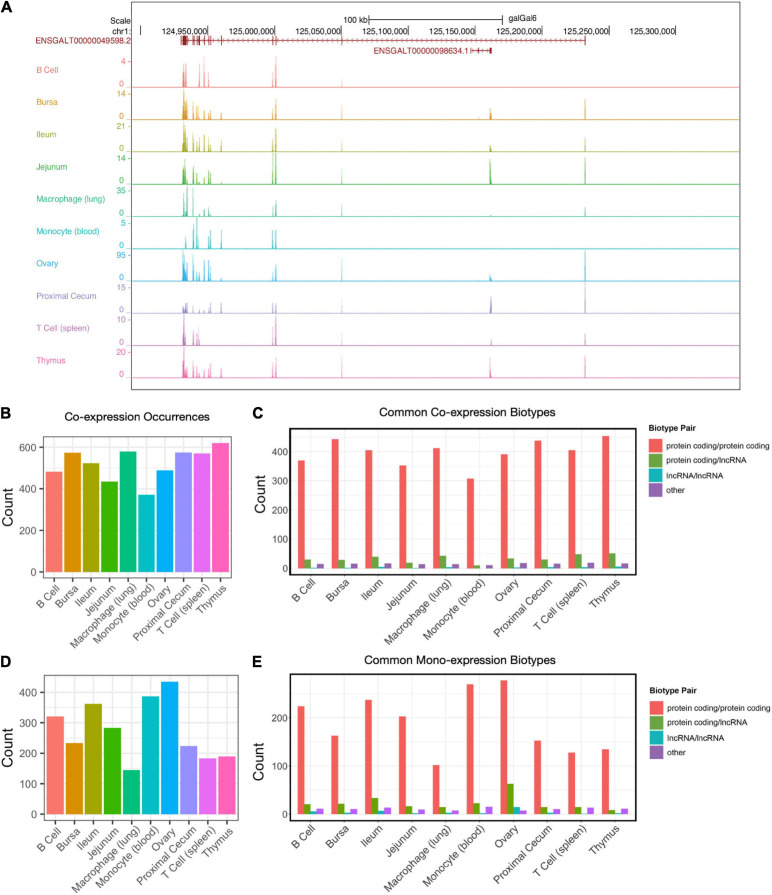
Forward–reverse strand co-expression. **(A)** Example of co-expressed transcripts on the forward and reverse strands. ENSGALT00000049598/*FRMPD4* (reverse strand) and ENSGALG00000098634 (forward strand) are overlapping in their genomic coordinates. **(B)** The number of co-expression occurrences in each tissue type. **(C)** Co-expression counts of protein-coding RNAs and lncRNAs. **(D)** The number of mono-expression occurrences in each tissue type. **(E)** Mono-expression of protein-coding RNAs and lncRNAs.

A subset of expression will occur within the same genomic coordinate range on strands opposite to one another. Co-expression of this kind can serve as a feedback mechanism to regulate the expression of one another, particularly between lncRNAs and protein-coding transcripts. An example of this is the expression of the protein-coding gene *FRMPD4* (ENSGALT00000049598) occurring on the strand opposite to the lncRNA gene ENSGALT00000098634 (Figure 4A). Co-expression was determined by locating genes whose 5′UTR-3′UTR sequences were overlapping by at least one base pair on opposite strands of one another. The number of co-expressed pairs ranged from 371 (monocyte) to 621 (thymus) (Figure 4B and Supplementary Table 4). The majority of pairs were both protein-coding genes for all cell and tissue types (range: 307–454) (Figure 4C). The next most common pairing was protein coding-lncRNA co-expression (range: 10–51). A small number of instances were lncRNA-lncRNA co-expression (range: 0–6). Also present were interactions between other biotypes (miRNA, pseudogene, misc_RNA, snoRNA, snRNA, scaRNA, rRNA, processed_pseudogene, IG_V_gene, Mt_rRNA, Mt_tRNA, ribozyme, sRNA) (range: 11–19).

Also of interest is mono-expression: when two genes occur within the same genomic coordinates range on opposite strands of one another, but only one of the genes is expressed. The number of mono-expressed pairs ranged from 145 (lung macrophage) to 435 (ovary) (Figure 4D). Similar to co-expressed genes, the most common pairing were pairs of protein-coding genes (range: 102–278), followed by protein coding-lncRNA mono-expression (range: 9–63), then lncRNA-lncRNA mono-expression (range: 1–15) (Figure 4E). There were also instances of mono-expression between other biotypes (miRNA, pseudogene, misc_RNA, snoRNA, snRNA, scaRNA, rRNA, processed_pseudogene, IG_V_gene, Mt_rRNA, Mt_tRNA, ribozyme, sRNA) (range: 8–16) (Supplementary Table 4).

Similar to co-expressed genes are head-to-head (H2H) genes. These genes are located on opposite strands and their TSSs are within 1 kb of each other. We detected 2,628 H2H genes in the *Gallus gallus* genome annotation. Out of these, 1,590 were detected within our cell/tissue samples (Supplementary Figure 6A). All of the H2H genes are between protein-coding genes. At the cell/tissue level, we detected between 812 [monocyte (blood)] and 1,146 (bursa) total H2H genes expressed. A small subset of these is unique to a single cell/tissue type, with a range between 2 [monocyte (blood)] and 44 [macrophage (lung)] (Supplementary Figures 6B,C and Supplementary Table 4). We also examined mono-expressed H2H genes (Supplementary Figures 6B,C) and detected between 1020 (bursa) and 1114 (ovary) H2H expressed genes at the cell/tissue level. Similar to co-expressed H2H genes, mono-expressed H2H genes have a small subset that is unique to each cell/tissue type, ranging between 3 (ileum) and 54 [T cell (spleen)] (Supplementary Table 4).

### DEG Analysis on Immune and Intestinal Samples

**FIGURE 5:**
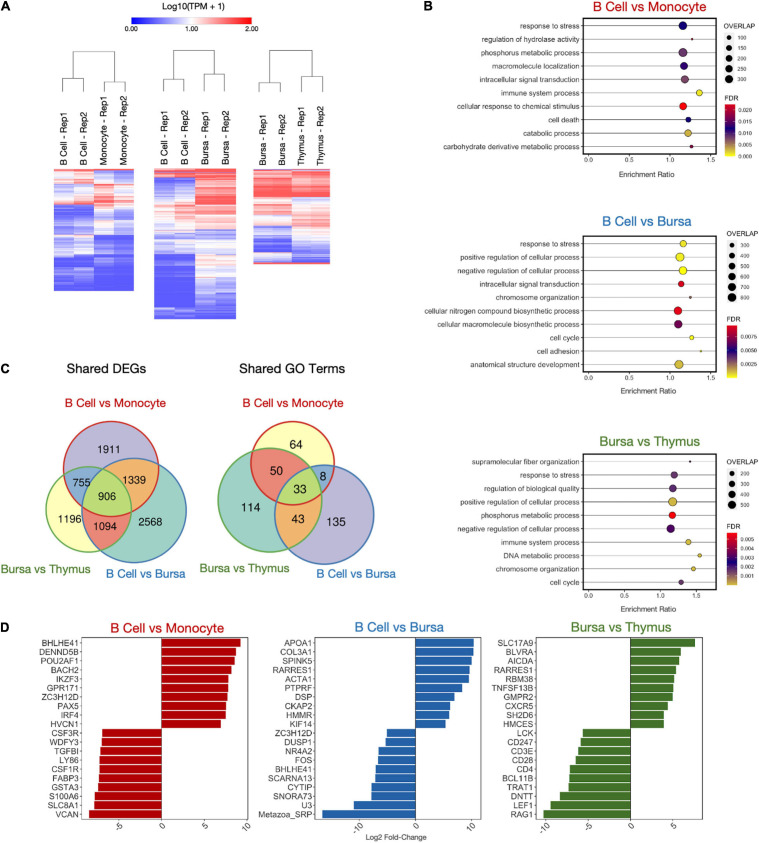
Differentially expressed gene (DEG) analysis on immune samples. **(A)** Heatmaps of DEGs in three cell type comparisons: B cells vs. monocytes (4911 DEGs), B cells vs. bursa tissue (5907 DEGs), bursa tissue vs. thymus tissue (3951 DEGs). Samples were clustered both by column and by row using Euclidean distance based on log-transformed TPM value. **(B)** Enriched GO biological process categories for each sample using weighted set cover filtering in WebGestalt. “Overlap” quantifies the number of DEGs present in that GO set. **(C)** The numbers of DEGs overlapping between-sample comparisons. **(D)** Log_2_ fold-change of top 10 upregulated and downregulated DEGs for each sample comparison.

In addition to determining genes and isoforms highly enriched for cell- or tissue-specific expression, we identified genes differentially expressed between related cells or tissues. Differentially expressed genes (DEGs) were computed for six cell/tissue type comparisons using Deseq2. Three of these comparisons were among immune cell samples. There were 4911, 5907, and 3951 DEGs for the comparisons B cells vs. monocytes, B cells vs. bursa tissue, and bursa tissue vs. thymus tissue, respectively (Figure 5A and Supplementary Table 5). A weighted set cover analysis in WebGestalt (Liao et al., 2019) was performed to reduce redundancy and find the most representative GO biological process categories among sample comparisons (Figure 5B). The GO category “response to stress” was the only category shared among all three comparisons. When we compare the DEGs across all three comparisons, we find that there is a subset of genes that are shared across multiple sample comparisons, however, there is a sizable number of genes unique to each tissue comparison (Figure 5C and Supplementary Table 5). This was also reflected in similarities between enriched GO categories, which shared 33 categories between all three comparisons. Additionally, we see unique sets of genes among the top 10 DEGs for each comparison (Figure 5D).

**FIGURE 6:**
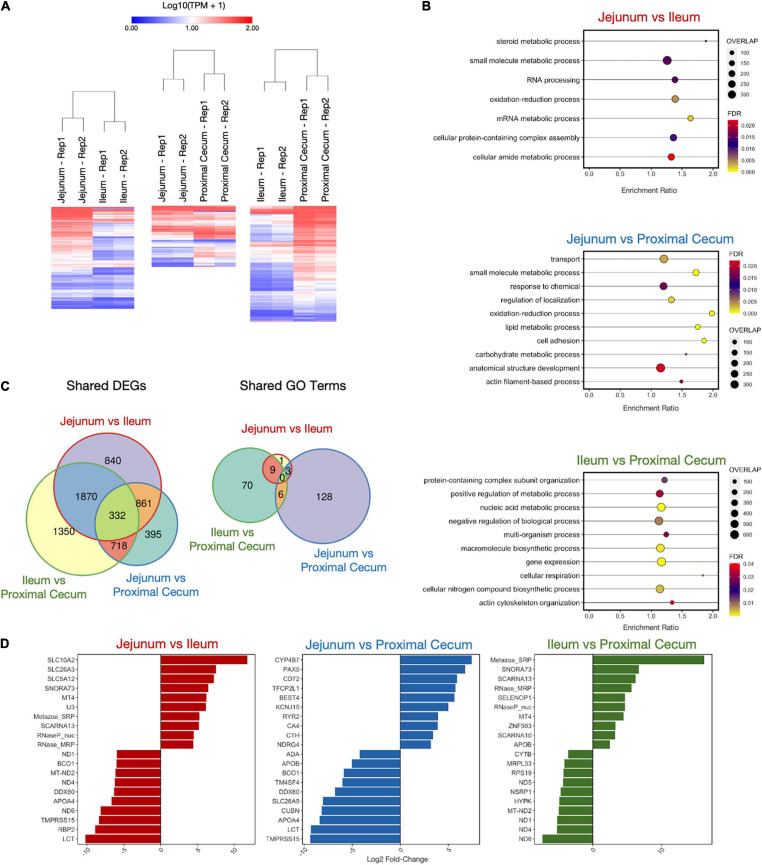
Differentially Expressed Gene (DEG) Analysis on Intestinal Samples. **(A)** Heatmaps of DEGs in three cell type comparisons: Jejunum vs. Ileum (3903 DEGs), Jejunum vs. Proximal Cecum (2306 DEGs), Ileum vs. Proximal Cecum (4270 DEGs). Samples were clustered both by column and by row using Euclidean distance based on log transformed TPM value. **(B)** Enriched GO Biological Process categories for each sample using weighted set cover filtering in WebGestalt. “Overlap” quantities the number of DEGs present in that GO set. **(C)** The numbers of DEGs overlapping between sample comparisons. **(D)** Log_2_ fold-change of top 10 upregulated and downregulated DEGs for each sample comparison.

Differentially expressed gene comparisons were also performed for three comparisons among intestinal samples. There were 3903, 2306, and 4270 DEGs for the comparisons of jejunum tissue vs. ileum tissue, jejunum tissue vs. proximal cecum tissue, and ileum tissue vs. proximal tissue, respectively (Figure 6A and Supplementary Table 6). A weighted set cover analysis was again performed (Figure 6B). There were no overlaps of enriched GO categories in the weighted set cover or among the sets of all GO terms enriched for each cell type, despite seeing 332 differentially expressed genes shared between all tissue comparisons (Figures 6B,C and Supplementary Table 6). Separating both DEG GO analyses, immune and intestinal, by upregulated and downregulated genes yields similar results (Supplementary Tables 7, 8). Among the sets of the top 10 differentially expressed genes for each tissue comparison, we observe the genes *APOA4* and *LCT* are present for tissue comparisons of jejunum vs. ileum and jejunum vs. proximal cecum (Figure 6D). Additionally, the tissue comparisons jejunum vs. ileum and ileum vs. proximal cecum share the five differentially expressed genes *MT-ND2, ND1, ND4, ND6*, and *SNORA73*.

Overrepresented KEGG pathways were also identified using WebGestalt for both of these immune and intestinal tissue comparisons. Each set of DEGs has a unique set of modified pathways, however, there are some overlaps between comparisons (Supplementary Figures 7A–C). In particular, in the immune system comparisons, the pathways “cell cycle” and “DNA replication” are enriched in the DEG sets for both the B cell vs. bursa and bursa vs. thymus comparisons (Supplementary Table 9). In the intestinal system comparisons, the “peroxisome” pathway is enriched in the DEG sets for jejunum vs. ileum and jejunum vs. proximal cecum. Additionally, the “spliceosome” pathway is enriched in the DEG sets for the jejunum vs. ileum and the ileum vs. proximal cecum. Out of all comparisons, the jejunum vs. proximal cecum has the most enriched pathways, many of which are involved in various metabolism and biosynthetic functions (Supplementary Figures 7D–F and Supplementary Table 10).

## Discussion

Side-by-side comparisons of transcriptomes were made for some of the immune cells and tissues, as well as intestinal tissues, to gain additional biological insight. B cells were compared to monocytes from peripheral blood, B cells with bursa, bursa with the thymus. The most significant (P-value) differentially expressed genes were highlighted in the results (Figure 5D). In the comparison between the monocytes and B cells, *CSF1R*, *GSTA3, LY86, S100A6, TGFβ1*, and *VCAN* were highly expressed in monocytes. Colony-stimulating factor-1 receptor (*CSF1R*) is a major stimulator of macrophage maturation from monocytes (Gan et al., 2020; Peng et al., 2020; Wu et al., 2020). Glutathione S-transferase α3 (*GSTA3*), for glutathione metabolism, is expressed in the macrophages against reactive oxygen species (ROSs) (McNeill et al., 2015). After phagocytosis of antigen or dead cells, macrophages release ROSs to destroy the ingested molecules through respiratory burst. Therefore, it is logical that monocytes have a higher expression of *GSTA3* to control the over-production of ROSs. Lymphocyte antigen 86 (*LY86*), also known as Myeloid Differentiating Protein-1 (*MD1*), activates toll-like receptors in innate immune cells (Candel et al., 2015). *S100A6* (calcyclin) has been implicated in cell differentiation and apoptosis (Donato et al., 2017). Transforming growth factor-β1 (*TGFβ1*) is produced by monocytes to regulate chemotaxis (McCartney-Francis et al., 1990; Sato et al., 2000). Versican (*VCAN*) is a chondroitin sulfate proteoglycan involved in cell proliferation (Zhang et al., 1998) and is produced by leukocytes to regulate inflammation (Wight et al., 2014). Due to the constant flux in monocyte development in the peripheral blood, it explains the higher expressions of *Ly86, S100A6, TGFβ1*, and *VCAN* in monocytes.

*DENND5B, HVCN1*, and *IKZF3*, and *POU2AF1, BACH2, and IRF4* expression were significantly upregulated in the B cells compared to monocytes. The role of DENN Domain Containing 5B (*DENND5B*) on B cells is unclear. B cell antigen receptor (*BCR*) signaling requires the internalization of BCR with Hydrogen Voltage-Gated Channel 1 (*HVCN1*) to regulate ROS production (Capasso et al., 2010). The Ikaros Family of Zinc-finger Protein-3 (*IKZF3*) is involved in early B cell development and its expression is increased progressively throughout B cell development (Ferreirós-Vidal et al., 2013). The POU Class 2 Homeobox Associating Factor 1 (*POU2AF1*) promotes B cell development and maturation (Zhao et al., 2008). BACH2 is involved in proliferation of B cells (Miura et al., 2018) and IRF4 is essential for lymphocyte function and involved in the development, affinity maturation, and terminal differentiation of B cells (Mittrücker et al., 1997).

*APOA1, PTPRF*, and *RARRES1* had higher expression in the B cells compared to bursa in this study. The bursa of Fabricius is a unique organ near the cloaca of the birds for B cell development and production (Glick et al., 1956). *APOA1*, short for Apolipoprotein A-1, is a major component in high-density lipoprotein (HDL) for lipid transport in the plasma. Interestingly, *APOA1* was one of the most abundant proteins identified in the bursa in early embryonic development (Korte et al., 2013). However, the bursas sampled for this study were more mature, which might explain that the gene expression was lower. It is not clear what the role of *APOA1* in B cells might be. *PTPRF*, short for Protein Tyrosine Phosphatase Receptor Type F, regulates Wnt signaling, which mediates B cell differentiation (Qiang et al., 2003; Gan et al., 2020). RARRES1 (Retinoic Acid Receptor Responder 1), also known as Tazarotene-induced gene 1 protein/RAR-responsive protein TIG1, facilitates retinoic acid synthesis from β-carotene (precursor of vitamin A) (Chung and Lo, 2007; Mihály et al., 2011). Vitamin A and retinoic acid are essential for B cell development and antibody production (Ross et al., 2011), as well as monocyte differentiation into macrophages (Gundra et al., 2017).

*BHLHE41*, short for Basic Helix-Loop-Helix Family Member E41, is a regulator of B cell development, which is consistent with our data showing that BHLHE41 is more expressed in bursa than in more mature peripheral B cells (Kreslavsky et al., 2017). The Cytohesin 1 interacting protein (*CYTIP*) regulates lymphocyte cell adhesion (Boehm et al., 2003), an important function of B cells. cFos is involved in immune receptor interaction (Bush and Bishop, 2008). The transcription factor, *NR4A2*, limits B cell activation when the secondary T cell signaling is absent (Tan et al., 2020).

We identified several non-coding RNAs with higher expression in the bursa than B cells, particularly *Metazoa_SRP, SCARNA13*, and *U3*. *Metazoa_SRP* encodes for a signal recognition particle RNA that is predominantly studied in archaea, bacteria, fungi, and protozoa species (Rosenblad et al., 2004; Dumesic et al., 2015). Little is known about the *Metazoa_SRP* gene in animals but it is thought to be involved in the translocation of RNA between the endoplasmic reticulo-membrane and cytosol (Shan and Walter, 2005) and post-translational transport of proteins to the ER (Abell et al., 2004). *SCARNA-13*, (small Cajal body-specific RNA-13), is a regulatory RNA. These small RNAs regulate gene expressions in the Cajal bodies by controlling small nucleolar RNA such as the *U3* (Richard et al., 2003; Allantaz et al., 2012).

In the comparison of the bursa and thymus DEGs, higher expressed genes in the thymus are essential genes for T cell and thymic development, such as *CD247* (Lundholm et al., 2010), *CD28* (Lenschow et al., 1996), *CD3E* (Call et al., 2002), *CD4* (Zhang et al., 2009; Zhu et al., 2009), *DNTT* (Su et al., 2004, 2005), *LCK* (Van Laethem et al., 2013), *LEF1* (Xing et al., 2019), *RAG1* (Xing et al., 2019), *TRAT1* (Mijušković et al., 2015), while the *BCL11B* transcription factor is involved in both B and T cell (Avram and Califano, 2014). Genes higher expressed in bursa included CXCR5, TNFSF13B, AICDA, and SH2D6 (or BLNK). CXCR5 plays an important role in the migration of B and T cells to secondary lymphoid organs (Legler et al., 1998) and has previously been shown to be highly expressed in bursa (Annamalai and Selvaraj, 2011). TNFSF13B is a cytokine that belongs to the tumor necrosis factor (TNF) ligand family and is also known as B cell-activating factor (BAFF). It is expressed in B cell lineage cells and has been shown to play an important role in the proliferation and differentiation of B cells (Mackay et al., 1999). AICDA, the gene coding for AID (activation-induced cytidine deaminase), is essential for immunoglobulin (Ig) gene somatic hypermutation (SHM) and class switch DNA recombination (CSR). AID expression is induced by activated B-cell CD40 signaling, critical for germinal center reaction (Park et al., 2009). Finally, SH2D6 or BLNK, functions as a central linker protein, downstream of the B-cell receptor (BCR). Activation leads to a multitude of signaling pathways and regulating biological outcomes of B-cell function and development (Ishiai et al., 1999).

In summary, many of these genes were mostly involved in cellular processes relating to differentiation and cell metabolism as well as basic functions of immune cells such as cell adhesion and signal transduction. This was to be expected, as there was no explicit immunological stimulus involved, the transcriptome rather represents the baseline activity at the time sampled. Nevertheless, it was notable that DEGs in the comparison between bursa and thymus that were upregulated in the thymus were related to T cell differentiation and maturation. On the other hand, genes differentially upregulated in B cell vs. bursa or bursa vs. thymus, are mostly involved in B cell development and differentiation, or activation. Genes differentially regulated in B cells and monocytes are involved in specific functions of the cell types.

While the chicken ileum was previously profiled (Kuo et al., 2017), the jejunum and cecum were not studied previously. We included the top ten genes of the differential expression analyses between tissue types based on levels of significance. Hierarchical clustering showed clear discrimination between the different parts of the intestine (Figure 6A). Of the 3,903 DEGs of the jejunal and ileal cells, the number of genes involved in steroid metabolism is the most different between jejunal and ileal tissues. Lipid metabolism of fat in the diet requires steroid biosynthesis of molecules such as bile acid from the pancreas into the small intestine (Dawson and Karpen, 2015). The bile acid emulsifies lipid molecules, which travel through the small intestine and allow fatty acids to be absorbed. Consistent with the observation in rats, absorption of steroidal hormones decreases throughout the small intestine (Nakayama et al., 1999). Unsurprisingly, bile acid absorption can be twice as high in the jejunum than in the ileum (Krag and Phillips, 1974; Aldini et al., 1996). This further confirms the higher lipid metabolism of the jejunum than the ileum in chickens (Tancharoenrat et al., 2014). Of the a06 DEGs of the jejunum and proximal cecum, the number of genes involved in the oxidation-reduction process, lipid metabolic process, and cell adhesion were the most different. The primary role of the jejunum is the digestion and absorption of nutrients. In contrast, the ceca are blind-sacs in the chicken intestine that play multiple roles in nutrients absorption including bacterial fermentation of small molecules and biosynthesis of short-chain fatty acids (propionic and butyric acids) (Clench, 1999). The proximal cecum contains the cecal tonsils, which are the largest gut-associated lymphoid tissues (GALT) in chickens that demonstrate protective immune responses in the intestinal tract (Heidari et al., 2015). Therefore, it is logical that the DEGs of these metabolic functions are more pronounced in the jejunum than in the cecum. Of the 4,270 DEGs between the ileum and cecum, the DEGs corresponding to cellular respiration were the most different. This could be expected as bacteria fermentation produces high levels of short-chain fatty acids in the cecum, which can be used as energy by the intestinal cells (Murugesan et al., 2014). Due to the relative size and metabolic demands of the ileum compared to the cecum, much energy is needed from aerobic respiration and mitochondrial electron transport to produce adequate energy in the ileum.

Interestingly, the comparisons of the jejunum to the ileum and the proximal cecum revealed differential expression of *LCT*, the gene encoded for lactase production, which is lower in the jejunum compared to that in the ileum and proximal cecum (Figure 6D). Since chickens are not mammals, the expression of the lactase gene is perplexing. The expression of the lactase gene in chickens has been debated in the past (Hamilton and Mitchell, 1924). Several hypotheses had been proposed about the presence of the lactase gene in chickens. The presence of the lactase gene could be due to (1) bacterial fermentation of lactase in the intestine, (2) evolutionary artifacts, or (3) improper annotation of the gene in chickens that could have the same sequence but functionally different in the chicken compared to mammals. An early study using based on the disappearance of lactase *in vitro* showed that lactase was assimilated in the crop but not in the proventriculus or the intestine (Plimmer and Rosedale, 1922). However, the assumption of the disappearance of lactase as evident of lactose digestion is flawed because it does not account for the microbial degradation of lactose. Later, molecular cloning confirmed lactase expression in the chicken intestinal tract as well as in mussel (Freund et al., 1997). Based on our sequencing results, we cannot conclude whether this is due to evolutionary artifacts, evolutionary converged traits with separate lineages, or genes with the same sequence but with completely different functions, or otherwise.

Among the DEGs from the intestinal tract, *APOA4* is responsible for lipid metabolism (Tso et al., 2004; Wang et al., 2020) and lipid-soluble vitamin metabolism such as retinoic acid (vitamin A) (Hebiguchi et al., 2015). Coincidentally, *APOA4* and retinoic acid-binding protein-2 (*RBP2*), and beta-carotene oxygenase 1 (*BCO1*) had higher expressions in the ileum compared to the jejunum. Mitochondrial NADH dehydrogenase (*MT-ND2*), NADH dehydrogenase-1 (*ND1*), NADH dehydrogenase-4 (*ND4*), NADH dehydrogenase-5 (*ND5*), and NADH dehydrogenase-6 (*ND6*) relate to the electron transport chain that generates cellular energy in the form of ATP through oxidative respiration (Weiss et al., 1991). These energy metabolic genes had higher expression in the ileum compared to the jejunum and in the proximal cecum compared to the ileum, suggesting higher energy production through aerobic respiration in these tissues. The Transmembrane Serine Protease 15 (*TMPRSS15*) is an enteropeptidase secreted from the pancreas that catabolizes trypsinogen to trypsin and chymotrypsinogen to procarboxypeptidase for protein digestion in the intestine (Zhang et al., 2009). Expression of *TMPRSS15* was higher in the ileum than the jejunum, suggesting the increasing rate of protein digestion throughout the small intestine. Consistent with a previous study on ion transport in the intestine (Wingate et al., 1973), several ion transporter genes, Solute Carrier Family 5 Member 12 (SLC5A12) for sodium and glucose co-transport, Solute Carrier Family 26 Member for chloride transport, and Solute Carrier Family 10 Member A2 for sodium and bile acid co-transport had higher expression in the jejunum than the ileum. Metallothionein-4 (MT4) is a tissue-specific binding protein for zinc and copper for sequestering the trace minerals from pathogens and regulating the intra- and extra-cellular concentrations (Sakulsak, 2012). Expression of *MT4* was higher in the jejunum in the current study.

Several of the DEGs highly expressed in the proximal cecum are involved in lipid metabolism, including *APOA4* and *APOB* (major components of lipoproteins) (Schianca et al., 2011), Beta-carotene Oxygenase-1 (*BCO1*) (lipid-soluble vitamin A metabolism), *CUBN* (lipoprotein endocytosis) (Christensen and Birn, 2002), and *SLC26A9* (bile metabolism) (Li et al., 2016). Surprisingly, *APOB* is higher expressed in the ileum than the proximal cecum. Adenosine deaminase (*ADA*) is involved in purine metabolism for nucleotide synthesis (Ikehara and Fukui, 1974) and is abundant in lymphocytes (Sakumi and Sekiguchi, 1989). Bacteria in the intestine are essential for vitamin absorption for the host (Ikehara and Fukui, 1974; LeBlanc et al., 2013). Therefore, it is consistent that *TM4SF4*, which is involved in thiamine (vitamin B1) metabolism, displayed higher levels of expression in the proximal cecum. The DEAD/DEAD-Box Helicase-60 is involved in innate immunity (Perčulija and Ouyang, 2019). The mucosal-associated lymphoid tissue (MALT) in the proximal cecum is the secondary lymphoid organ of the chicken, and the cecum houses microbiota that regulates metabolism (Polansky et al., 2016). This could explain the higher expression of the DEAD/DEAD-Box Helicase-60 (*DDX60*), Adenosine deaminase (*ADA*), and Liver Enriched Antimicrobial Peptide 2 (LEAP2) in the proximal cecum.

The potassium inwardly rectifying channel subfamily J member 15 (*KCNJ15*) (Yuan et al., 2015), ryanodine receptor 2 (*RYR2*) (Jiang et al., 2004), and the bestrophin family anion channel (*BEST4*) (Fischmeister and Hartzell, 2005) for ion exchanges were upregulated in the jejunum compared to the proximal cecum. The carbonic anhydrase (*CA4*) utilizes zinc to produce carbonic acid for maintaining acid-base balance (Sly and Hu, 1995). Glutathionase (*CTH)* utilizes glutathione for antioxidant production against reactive oxygen species (ROS) (McBean, 2017). Since the jejunum is responsible for nutrient absorption, whereas the cecum is a blind sac that is involved in immunity, it is conceivable that these genes are higher expressed in the jejunum compared to the proximal cecum. In addition, two transcription factors were upregulated in the jejunum: transcription factor CP2 like 1 (*TFCP2L1*) and paired box family of transcription factor (*PAX5*). The former is involved in epithelial cells’ development consistent with the high turnover of intestinal epithelial cells (Werth et al., 2017). However, the latter is involved in B cell development (Nutt et al., 1999). *CD72* regulates B cell development and signaling and it showed higher levels of expression in the jejunum compared to the proximal cecum (Kumanogoh et al., 2000). *CYP4B7* belongs to the cytochrome P450 family detoxification enzyme (Alber et al., 2020). The higher expression in the jejunum is consistent with its digestive functions. Three trace mineral-related genes had higher expression in the ileum than the proximal cecum: selenoprotein (*SELENOP1*), metallothionein-4 (*MT4*), and zinc finger protein 593 (*ZNF593*). N-myc downregulated gene family (*NDRG4*) regulates smooth muscle cells (Qu et al., 2016). Similar to *NDRG4*, *ZNF593* regulates muscle cell differentiation (Lynch et al., 2019). Consistently, *NDRG4* and *ZNG593* are less expressed in the proximal cecum because the primary function of the cecum is thought to be related to modulate immunity and metabolism through the microbiota; whereas the jejunum and ileum are primarily responsible for digestion and absorption of nutrients that require contraction of smooth muscles during peristalsis.

In summary, we were able to correlate most of the differential expressed genes in the intestine to mostly metabolic processes related to nutrient digestion and absorption. Several genes in the distal part of the intestine were particularly implicated in vitamin metabolism. This was not surprising because vitamin metabolism requires the microbiota, which is more abundant in the distal intestines. Genes involved in energy metabolism are also abundant in the cecum, which suggests that microbial contribution of energy production in the intestine is especially important.

In the current study, whole transcriptome RNA-seq of immune, intestinal, and reproductive cells and tissues were sequenced. The Ensembl chicken annotation release 98 (GRCg6a, genome-build-accession NCBI:GCA_000002315.5), contains 16,779 protein-coding genes, 7,577 non-coding genes, and 39,288 gene transcripts. Of the non-coding genes, 5,504 were long non-coding genes; 10,301 lncRNAs are annotated when considering Jehl et al. (2020). From 10 diverse cell and tissue types, we recovered 73% of annotated genes and 63% of known transcripts. Of annotated genes, 90% of coding genes are expressed in the 10 cell and tissue types studied here, while only 36% of annotated lncRNAs are expressed. The potential regulatory role of lncRNAs may explain the limited expression, and suggest a more cell- or tissue-specific role. We found that biosamples often expressed hundreds of cell- or tissue-specific coding genes and lncRNAs. While many genes are commonly expressed in multiple samples, we also determined that over 500 isoforms of genes are uniquely expressed. Each cell and tissue type only expressed an average of 1–2 gene isoforms; however, each biosample type had at least one gene with 11 or more isoforms expressed in the cell or tissue type. We did not attempt to annotate novel genes base due to our current sequence depth per sample. Analysis of differentially expressed genes revealed biological processes that are consistent with a function in the cells or tissues of interest. Continued investigation of these genes should further our understanding of disease susceptibility/resistance, feed conversion, and egg production. Collectively, these data provide a deeper understanding of the chicken transcriptome in a cell- and tissue-specific manner. We have provided lists of unique transcripts, genes with high isoform count, sense-antisense co-expression pairs, and differentially expressed genes in our Supplementary Tables as a resource to the community. Additional samples from the FAANG and greater community will continue to advance efforts toward a comprehensive catalog of the chicken transcriptome.

## Data Availability Statement

The datasets presented in this study can be found in online repositories. The names of the repository/repositories and accession number(s) can be found below: Gene Expression Omnibus (GEO) – GSE166257.

## Ethics Statement

The animal study was reviewed and approved by Western University of Health Sciences, Pomona, California (WesternU) Institutional Animal Care and Use Committee.

## Author Contributions

EO performed data analysis, figure generation, and manuscript writing. TN performed sample isolation and preparation, data interpretation, and manuscript writing. PC, LG, and PS contributed to sample isolation and preparation. ST led the tissue collection. YD and RH conceived the study, guided data analysis, contributed to data interpretation, and manuscript writing. All authors contributed to the article and approved the submitted version.

## Conflict of Interest

The authors declare that the research was conducted in the absence of any commercial or financial relationships that could be construed as a potential conflict of interest.

## Footnotes

1 https://software.broadinstitute.org/morpheus

2 http://www.fragencode.org/lnchickenatlas.html

## Supplementary Material

Supplementary Figure 1 Pairwise correlations. Pearson correlation plot of all genes in a sample for each pairwise comparison generated with pcaExplorer. Plots use a subset of 1000 genes and use log_2_ normalized gene counts for plot axes and values, respectively.

Supplementary Figure 2 Gene and isoform expression specificity. **(A)** Top 2000–4000 most specific genes. **(B)** The 1000 least specific genes. **(C)** The top 1000 most specific isoforms. **(D)** 1000 least specific isoforms. Rows sorted by Euclidean distance. **(C,D)** Isoforms have been filtered for genes that have a TPM of at least 0.5 in at least two cell types. Matrix entries that have no expression of that isoform’s gene are colored black.

Supplementary Figure 3 Extended lncRNA analysis. **(A)** Total lncRNA counts using the extended lncRNA annotation. **(B)** Cell/tissue-type specific lncRNA counts using the extended lncRNA annotation. **(C)** Top 1000 most specific lncRNAs for the extended lncRNA annotation.

Supplementary Figure 4 Isoform histograms. **(A)** Histogram of isoform counts per gene for tissues not included in Figure 3B. The cutout plot in the upper-right corner is a zoomed-in section for 4+ isoforms per gene. **(B)** GO biological process and molecular function analysis for genes with 4+ isoforms.

Supplementary Figure 5 Extended isoform visualization. **(A)** Additional sashimi plots from *PDGFRB* in Figure 3D. **(B)** Browser shot of the same annotated isoform for the gene *PDGFRB*.

Supplementary Figure 6 H2H transcripts. **(A)** Percent of total co-expressed H2H transcripts (2,628) detected across all cell/tissue types. **(B)** Total H2H transcript counts by cell/tissue type. **(C)** Cell/tissue type-specific H2H transcript count.

Supplementary Figure 7 Enriched pathways. **(A–C)** Enriched KEGG pathways for the immune comparisons in Figure 5 (FDR < 0.05). **(D–F)** Enriched KEGG pathways for the intestinal comparisons in Figure 6 (FDR < 0.05).

Supplementary Table 1 Sequencing read counts.

Supplementary Table 2 Cell type specific transcripts and lncRNAs (related to Figure 2). (Sheet 1) Protein coding RNAs. (Sheet 2) lncRNAs. (Sheet 3) Extended lncRNA analysis transcripts.

Supplementary Table 3 Isoforms (related to Figure 3). (Sheet 1) Isoforms from specificity plot (Figure 3A). (Sheet 2) Genes with 4+ isoforms per tissue/cell type (cutouts in Figure 3C and Supplementary Figure 4A).

Supplementary Table 4 Co-expressed and H2H pairs (related to Figure 4 and Supplementary Figure 6). (Sheet 1) Co-expressed transcript pairs. (Sheet 2) Mono-expressed transcript pairs. (Sheet 3) Co-expressed H2H transcript pairs. (Sheet 4) Mono-expressed H2H transcript pairs.

Supplementary Table 5 Immune differential expression and GO results (related to Figure 5). (Sheet 1) B cell vs. monocyte DESeq2 results. (Sheet 2) B cell vs. bursa DESeq2 results. (Sheet 3) Bursa vs. thymus DESeq2 results. (Sheet 4) B cell vs. monocyte WebGestalt (GO) results. (Sheet 5) B Cell vs. bursa WebGestalt (GO) results. (Sheet 6) Bursa vs. thymus WebGestalt (GO) results.

Supplementary Table 6 Intestinal differential expression and GO results (related to Figure 6). (Sheet 1) Jejunum vs. ileum DESeq2 results. (Sheet 2) Jejunum vs. proximal cecum DESeq2 results. (Sheet 3) Ileum vs. proximal cecum DESeq2 results. (Sheet 4) Jejunum vs. ileum WebGestalt (GO) results. (Sheet 5) Jejunum vs. proximal cecum WebGestalt (GO) results. (Sheet 6) Ileum vs. proximal cecum WebGestalt (GO) results

Supplementary Table 7 Separate up/down regulated immune DEGs. (Sheet 1) B cell vs. monocyte WebGestalt (GO) results – UPREGULATED DEGs ONLY. (Sheet 2) B cell vs. monocyte WebGestalt (GO) results – DOWNREGULATED DEGs ONLY. (Sheet 3) B cell vs. bursa WebGestalt (GO) results – UPREGULATED DEGs ONLY. (Sheet 4) B cell vs. bursa WebGestalt (GO) results – DOWNREGULATED DEGs ONLY. (Sheet 5) Bursa vs. thymus WebGestalt (GO) results – UPREGULATED DEGs ONLY. (Sheet 6) Bursa vs. thymus WebGestalt (GO) results – DOWNREGULATED DEGs ONLY.

Supplementary Table 8 Separate up/down regulated intestinal DEGs. (Sheet 1) Jejunum vs. ileum WebGestalt (GO) results – DOWNREGULATED DEGs ONLY. (Sheet 2) Jejunum vs. proximal cecum WebGestalt (GO) results – UPREGULATED DEGs ONLY. (Sheet 3) Jejunum vs. proximal cecum WebGestalt (GO) results – DOWNREGULATED DEGs ONLY. (Sheet 4) Ileum vs. proximal cecum WebGestalt (GO) results – UPREGULATED DEGs ONLY. (Sheet 5) Ileum vs. proximal cecum WebGestalt (GO) results – DOWNREGULATED DEGs ONLY.

Supplementary Table 9 Immune pathway results. (Sheet 1) B cell vs. monocyte WebGestalt (pathway) results. (Sheet 2) B Cell vs. bursa WebGestalt (pathway) results. (Sheet 3) Bursa vs. thymus WebGestalt (pathway) results.

Supplementary Table 10 Intestinal pathway results. (Sheet 1) Jejunum vs. ileum WebGestalt (pathway) results. (Sheet 2) Jejunum vs. proximal cecum WebGestalt (pathway) results. (Sheet 3) Ileum vs. proximal cecum WebGestalt (pathway) results.
