# Supplementary figures and images for: Transcriptomes of an Array of Chicken Ovary, Intestinal, and Immune Cells and Tissues
Source: Front Genet. 2021 Jun 30;12:664424. doi: 10.3389/fgene.2021.664424 (PMC8278112; doi:10.3389/fgene.2021.664424)

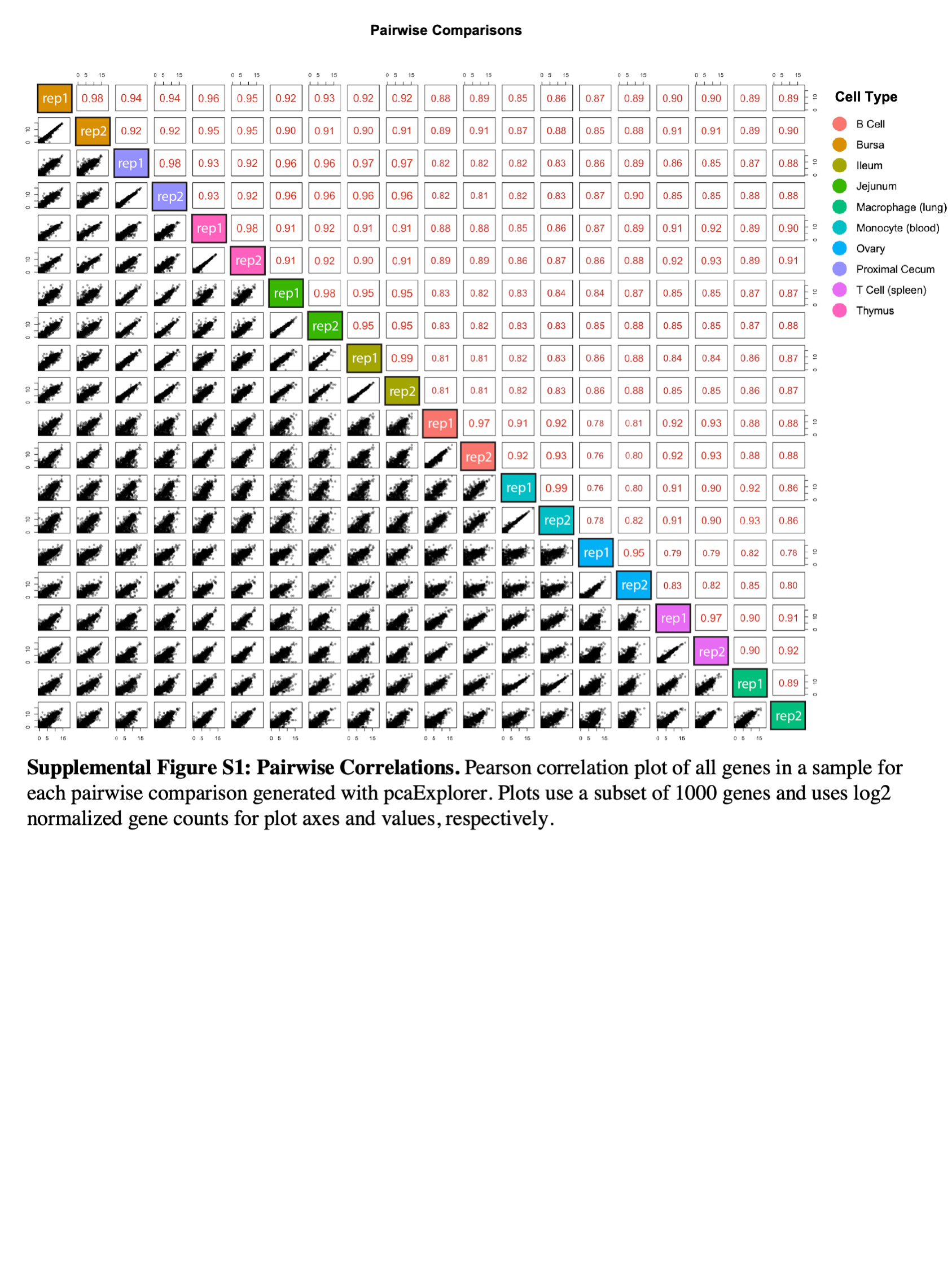

Supplement: Supplementary Figure 1 — Pairwise correlations. Pearson correlation plot of all genes in a sample for each pairwise comparison generated with pcaExplorer. Plots use a subset of 1000 genes and use log2 normalized gene counts for plot axes and values, respectively. [file Image_1.tiff]

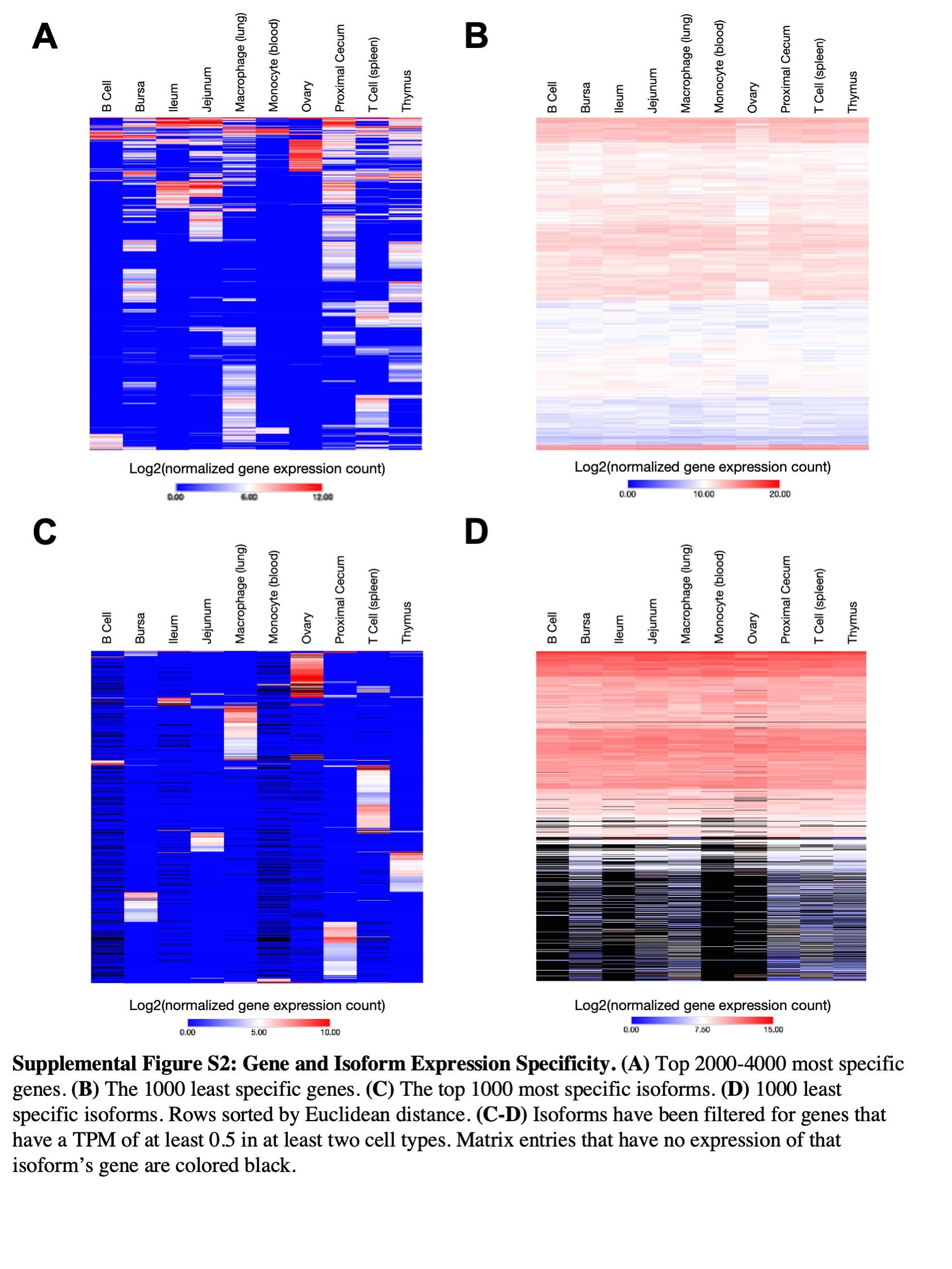

Supplement: Supplementary Figure 2 — Gene and isoform expression specificity. (A) Top 2000–4000 most specific genes. (B) The 1000 least specific genes. (C) The top 1000 most specific isoforms. (D) 1000 least specific isoforms. Rows sorted by Euclidean distance. (C,D) Isoforms have been filtered for genes that have a TPM of at least 0.5 in at least two cell types. Matrix entries that have no expression of that isoform’s gene are colored black. [file Image_2.tiff]

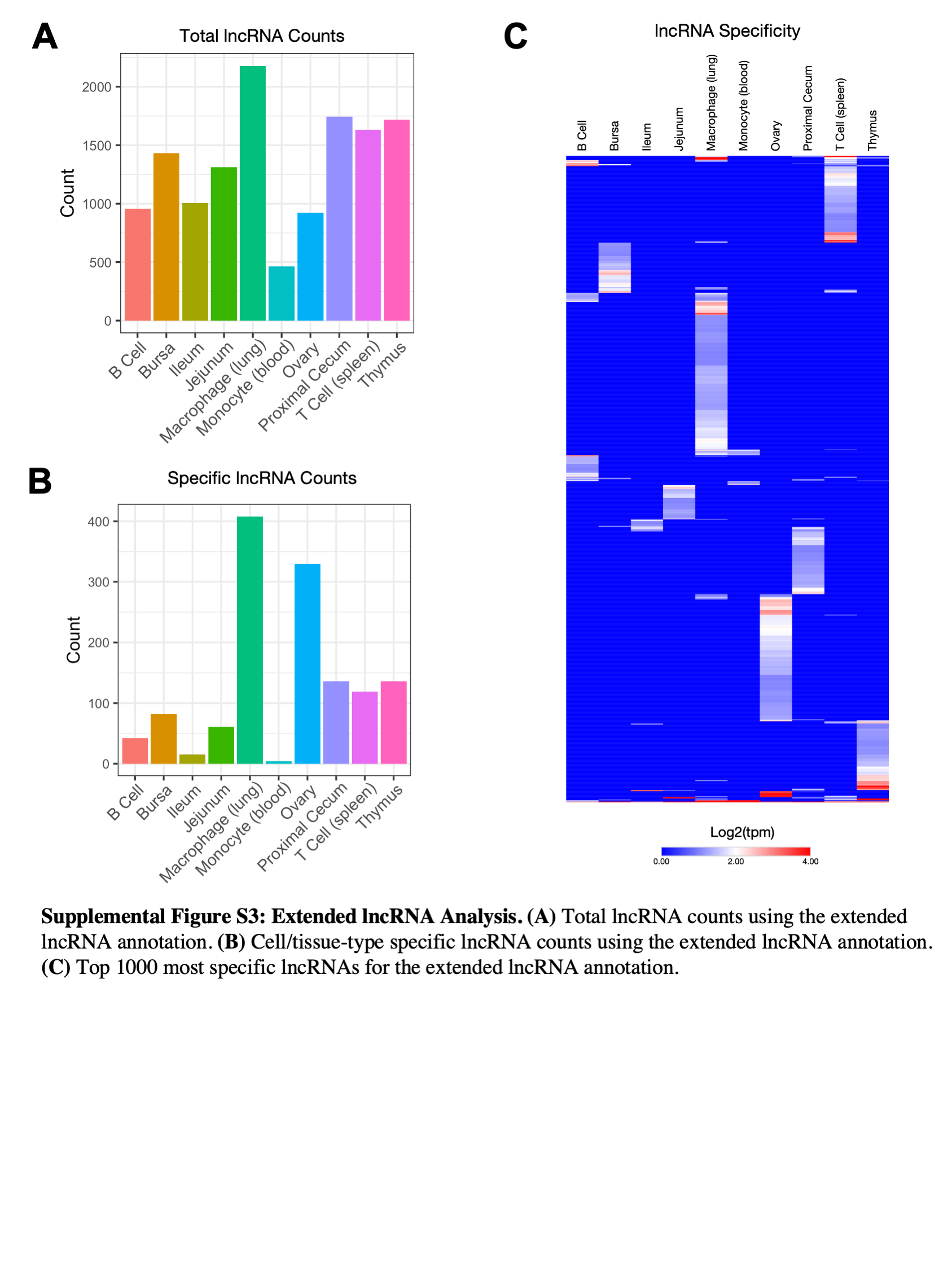

Supplement: Supplementary Figure 3 — Extended lncRNA analysis. (A) Total lncRNA counts using the extended lncRNA annotation. (B) Cell/tissue-type specific lncRNA counts using the extended lncRNA annotation. (C) Top 1000 most specific lncRNAs for the extended lncRNA annotation. [file Image_3.tiff]

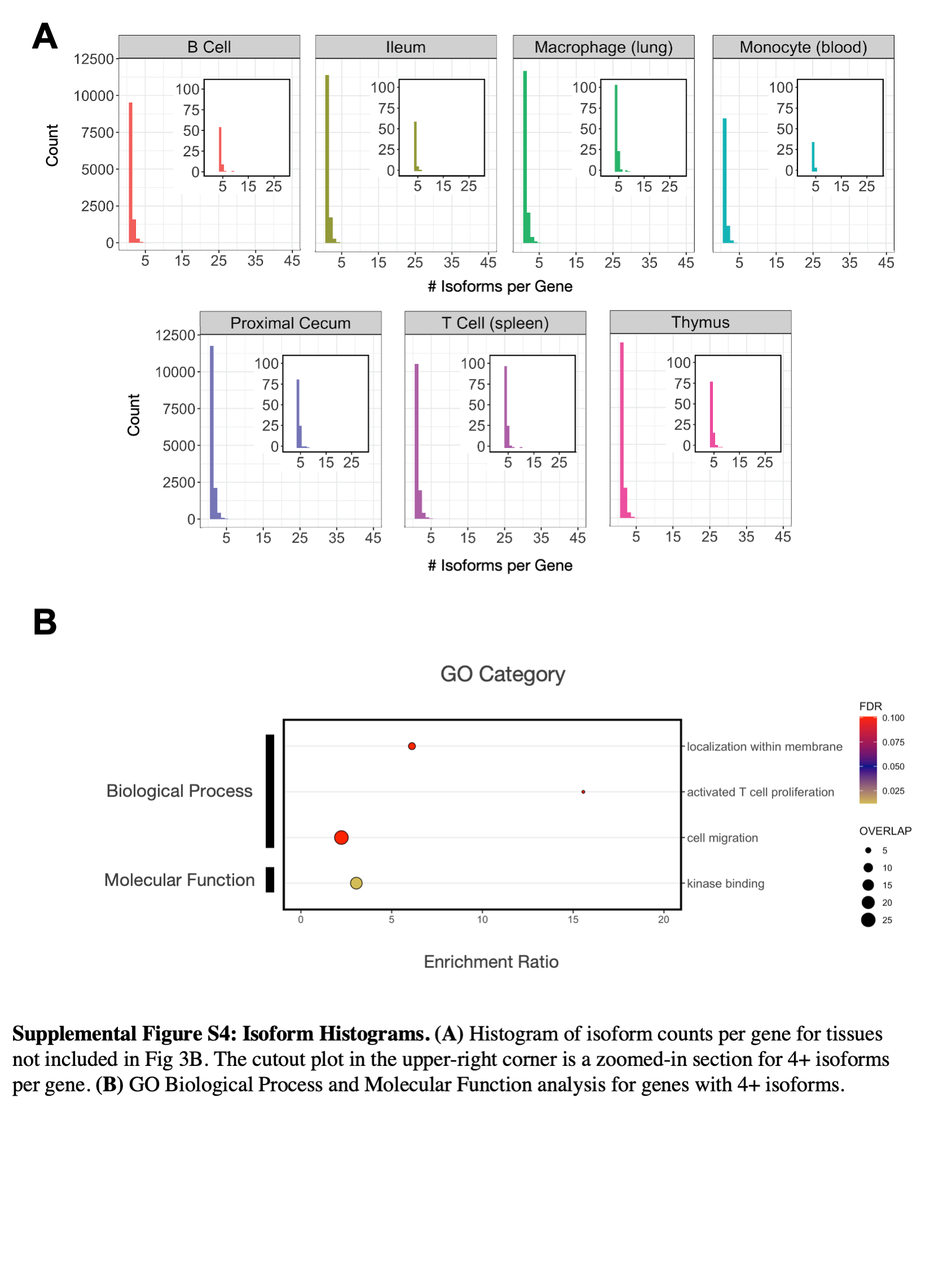

Supplement: Supplementary Figure 4 — Isoform histograms. (A) Histogram of isoform counts per gene for tissues not included in Figure 3B. The cutout plot in the upper-right corner is a zoomed-in section for 4+ isoforms per gene. (B) GO biological process and molecular function analysis for genes with 4+ isoforms. [file Image_4.tiff]

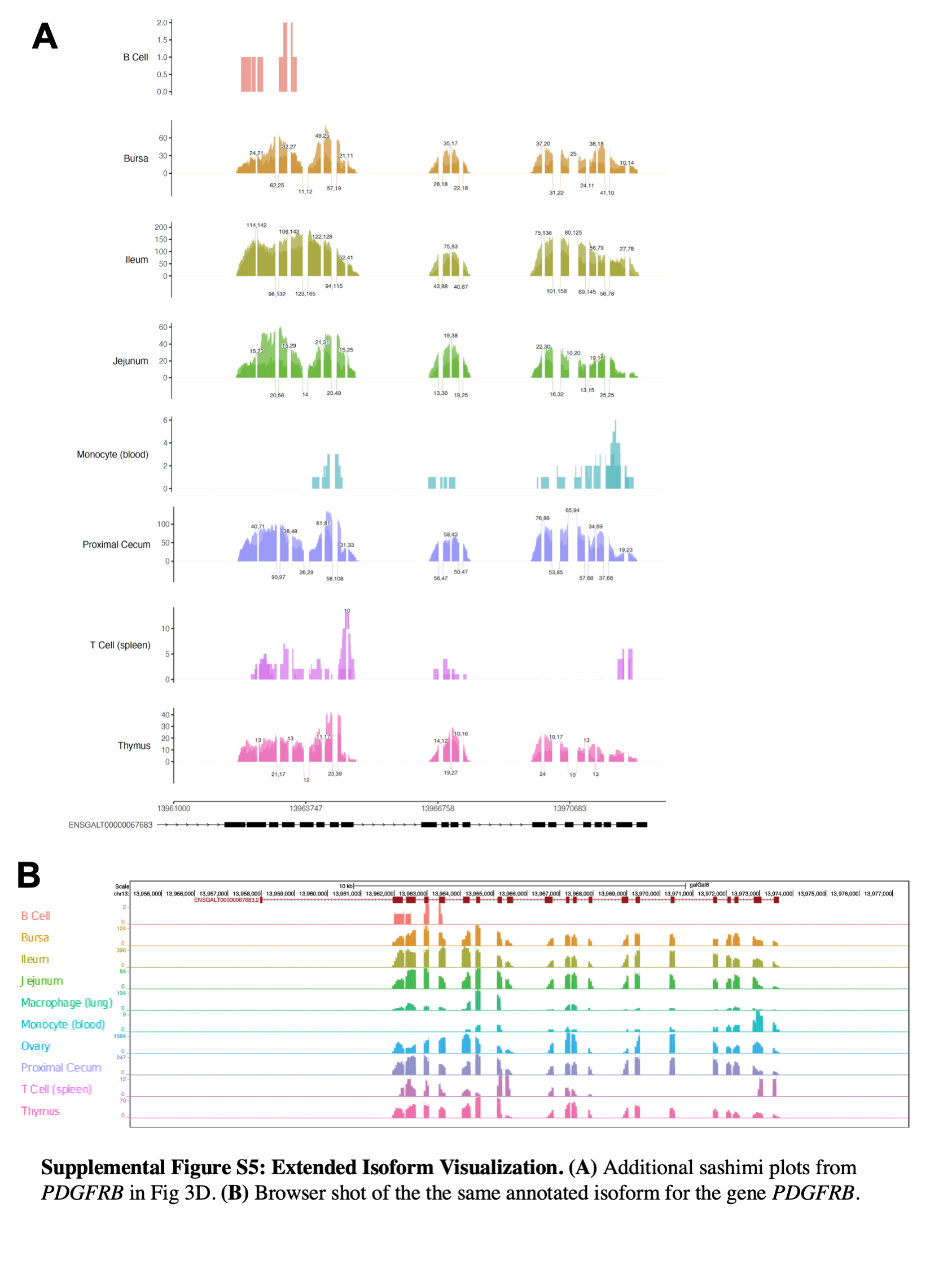

Supplement: Supplementary Figure 5 — Extended isoform visualization. (A) Additional sashimi plots from PDGFRB in Figure 3D. (B) Browser shot of the same annotated isoform for the gene PDGFRB. [file Image_5.tiff]

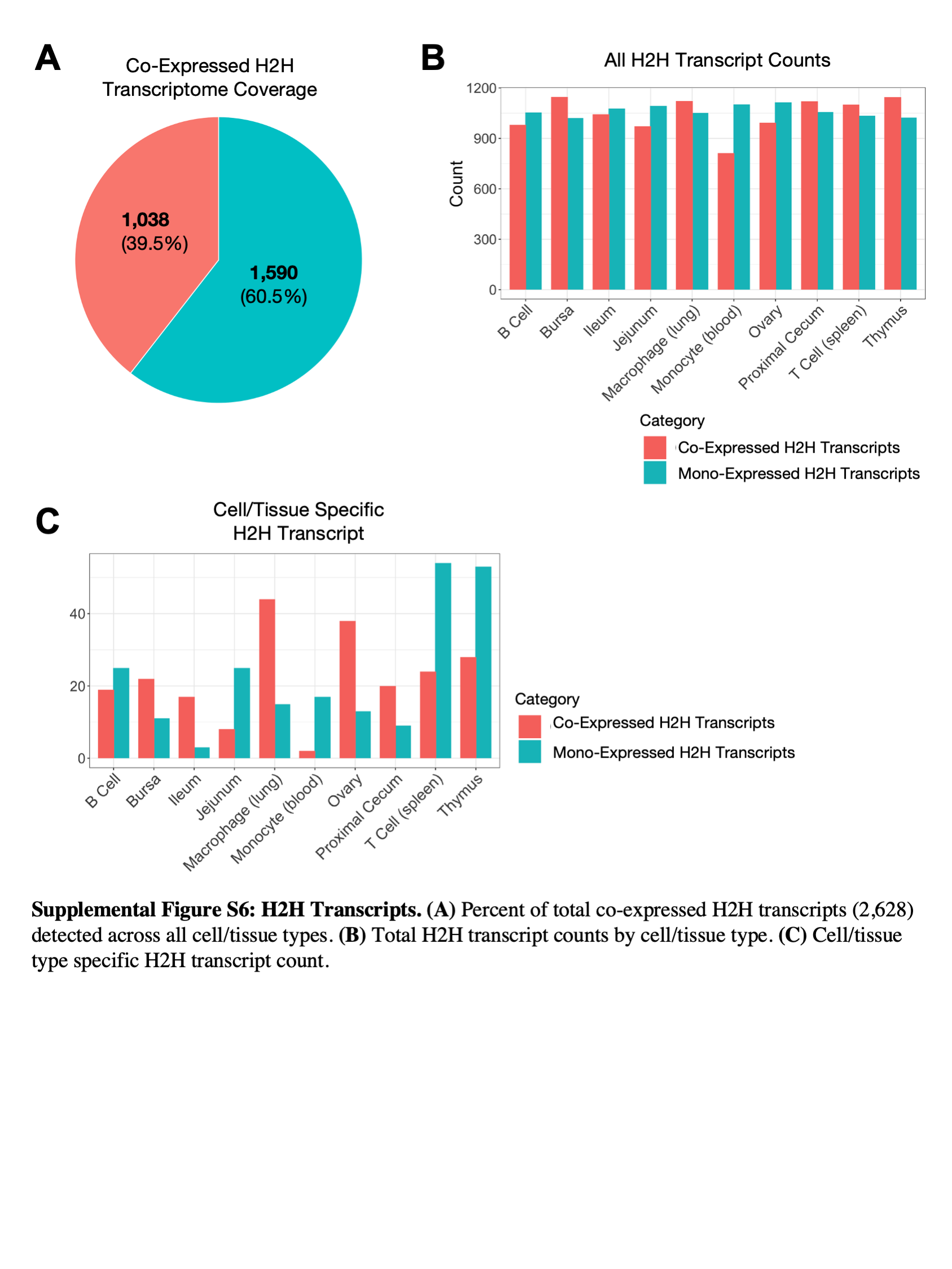

Supplement: Supplementary Figure 6 — H2H transcripts. (A) Percent of total co-expressed H2H transcripts (2,628) detected across all cell/tissue types. (B) Total H2H transcript counts by cell/tissue type. (C) Cell/tissue type-specific H2H transcript count. [file Image_6.tiff]

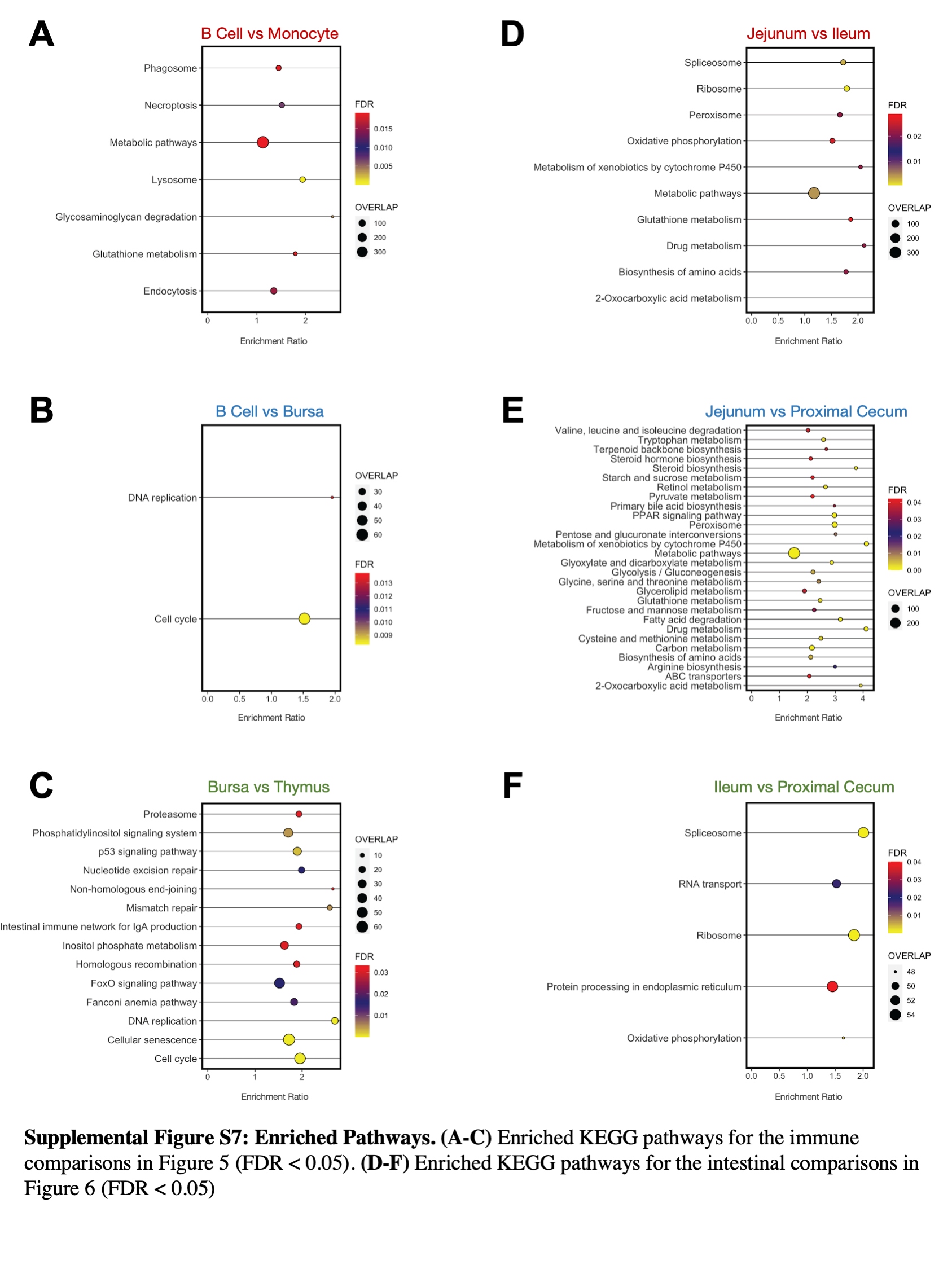

Supplement: Supplementary Figure 7 — Enriched pathways. (A–C) Enriched KEGG pathways for the immune comparisons in Figure 5 (FDR < 0.05). (D–F) Enriched KEGG pathways for the intestinal comparisons in Figure 6 (FDR < 0.05). [file Image_7.tiff]
